# Telephone-Based Mental Health Promotion for Rural Women in Brazilian Agrarian Reform Communities: Pre-Post Pilot Study

**DOI:** 10.2196/79473

**Published:** 2026-04-07

**Authors:** Jaqueline Lemos de Oliveira, Janaína Cristina Pasquini de Almeida, Jamila Souza Gonçalves, Adriana Moreira Alves, Antonio José Correa de Pauli, Denise Saint - Arnault, Jacqueline de Souza

**Affiliations:** 1 Department of Maternal-Infant and Psychiatric Nursing, College of Nursing, Universidade de São Paulo, Av. Dr. Enéas de Carvalho Aguiar, 419, São Paulo, São Paulo, 05403-000, Brazil, 55 1130617606; 2 Psychiatric Nursing and Human Sciences Program, College of Nursing at Ribeirão Preto, Universidade de São Paulo, Ribeirão Preto, São Paulo, Brazil; 3 Instituto Federal de Educação, Ciência e Tecnologia de Minas Gerais, Passos, Minas Gerais, Brazil; 4 Department of Health Behavior and Clinical Sciences, School of Nursing, University of Michigan, Ann Arbor, Michigan, United States

**Keywords:** women, mental health, health promotion, social vulnerability, rural population, psychosocial intervention

## Abstract

**Background:**

Women living in rural agrarian reform communities face intersecting challenges related to social, economic, racial, and gender vulnerabilities, which significantly increase their likelihood of developing physical and mental health problems. Despite the potential of telephone-based interventions to promote mental health, there is a lack of studies assessing their feasibility and effectiveness among underserved populations in Brazil.

**Objective:**

This study aimed to assess the feasibility and effectiveness of a telephone-based intervention on mental health outcomes among women living in a rural agrarian reform community in Brazil.

**Methods:**

We conducted a descriptive, prospective pilot study with a pretest and posttest design. Data were collected at 3 time points: baseline, 1 week, and 1 month after the intervention. The outcomes assessed included quality of life, social support, self-efficacy, and common mental disorder symptoms. Nonparametric tests were used to analyze the data. The intervention consisted of 3 phone calls supported by a workbook, with content based on cognitive behavioral and psychiatric nursing principles.

**Results:**

Of the 31 women enrolled, 23 (74.2%) completed all 3 phone-based sessions. There was a significant reduction in common mental disorder symptoms (Kendall *W*=0.280; *P*=.002), particularly in the somatic domain (*P*=.02). Moreover, participants reported improved perceptions of the physical domain of quality of life (Kendall *W*=0.131; *P*=.049). All women rated the intervention positively, with more than half emphasizing its practical usefulness.

**Conclusions:**

The telephone-based intervention was feasible and showed promising results in improving mental health outcomes among women in a rural setting. These findings support integrating low-intensity, remote psychosocial strategies into primary health care, especially those led by nurses, to increase access to mental health promotion for vulnerable populations.

## Introduction

Mental health care has been advocated for as a priority for developing public policies globally, and primary health care (PHC) is recognized as a key channel for expanding access to this care [1]. This is because PHC services are considered accessible to the community. They work from a comprehensive, intersectoral, interdisciplinary perspective, aiming to coordinate preventive, promotive, therapeutic, and rehabilitative actions according to users’ needs [2,3].

In this context, actions aimed at mental health care have been the focus of many studies, with an emphasis on the need to think about new strategies to expand such actions [4,5]. The telephone has emerged as a valuable tool for achieving these goals because it is a useful and easily accessible means of communication. It helps fill gaps in face-to-face care resulting from an overloaded health system, a lack of interprofessional collaboration, inadequate physical space, reduced resources, and user stigma [4-7].

Studies on the effectiveness of interventions delivered via telephone have been carried out in different countries and with diverse populations [6-8]. These interventions have proven beneficial, leading to significant reductions in depressive symptoms, worry, posttraumatic stress disorder, and anxiety; improvements in physical health; reduction in suicidal ideation; and greater satisfaction with treatment reported by participants [6-8]. However, most of these studies have focused on reducing symptoms among individuals with established diagnoses. Comparatively, less attention has been given to promoting mental health among socially vulnerable populations who may not have formal diagnoses or regular access to care.

In low- and middle-income countries, particularly in rural settings, structural barriers such as geographic isolation, limited availability of specialized services, socioeconomic disadvantage, and gender inequalities may further restrict access to mental health care. In Brazil, rural populations, including those living in rural agrarian reform communities, often face overlapping vulnerabilities related to poverty, labor instability, limited infrastructure, and restricted access to health care, education, and social services [9-12].

The Sustainable Development Goals include mental health and well-being, gender equality, and reducing inequality. Developing strategies for vulnerable populations is essential to achieving these goals. In this regard, women living in rural agrarian reform communities require special attention, as they face intersecting challenges related to social, economic, gender, racial, and other issues, which greatly increases their likelihood of developing physical and mental health problems [10-12].

Therefore, implementing accessible interventions that address both subjective and relational aspects of mental health for women living in rural agrarian reform communities is of paramount importance to expand access to mental health care, especially in the context of PHC. This study aimed to assess the feasibility and effectiveness of a telephone-based intervention on the mental health outcomes of women living in a rural agrarian reform community in Brazil.

## Methods

### Study Design

This is a single-group, nonrandomized preintervention and postintervention study, conducted in accordance with the Template for Intervention Description and Replication (TIDieR) recommendations. TIDieR is a tool used to describe the components of an intervention to facilitate replication and consistent implementation [13], and the TREND (Transparent Reporting of Evaluations with Nonrandomized Designs) statement provides guidance for reporting nonrandomized intervention studies. No control group was included due to the exploratory and feasibility nature of the study.

The study was conducted in a rural agrarian reform community in a municipality in the countryside of São Paulo state between August and December 2021.

### Participants and Data Collection

The study population consisted of adult women registered at the basic health unit (BHU) in the aforementioned rural agrarian reform community (approximately 1000). To be eligible, participants had to be literate, aged at least 18 years, and present at the BHU during data collection. As a formative feasibility study, no formal sample size calculation was performed.

Data collection was carried out by 2 graduate-level nurses who received training to administer the instruments listed in this research. This collection took place at 3 different points: baseline (before program implementation); first follow-up (1 week after program completion); and second follow-up (1 month after program completion).

A questionnaire was administered to assess participants’ socioeconomic profiles, and the following psychometric scales were applied: the World Health Organization Quality of Life abbreviated version (WHOQOL-BREF); Sarason Social Support Questionnaire (SSQ); the General Perceived Self-Efficacy Scale (GSE); and the Self-Reporting Questionnaire-20 (SRQ-20).

The WHOQOL-BREF contains 26 questions—two are general about quality of life (QoL) and the others represent each of the 24 facets divided into four domains: (1) physical, (2) psychological, (3) social relationships, and (4) environment. The questions are rated on a Likert scale from 1 to 5. The instrument contains 3 questions with inverted scores. The final score falls within a range of 4 to 20 and can be converted to a scale of 0 to 100 using the syntax developed by the authors [14]. Higher scores reflect better perceived QoL [14]. The instrument has been validated for use with the Brazilian population [14].

The SSQ consists of 27 questions addressing various situations where social support might be important for an individual. Respondents list up to 9 people per question and rate their satisfaction with the support in each situation. The mean of the scores measures both the size of the social network and satisfaction with the support [15]. This study used the SSQ’s short version (SSQ6), which includes 6 items and has been translated and validated for use with the Brazilian population [15].

The GSE, validated for the Brazilian population, assesses an individual’s general sense of perceived self-efficacy, aiming to predict how well they cope with daily challenges and adapt after stressful life events [16]. It consists of 10 items scored on a scale from 1 to 4, and the total score is calculated by summing the items, ranging from a minimum of 10 to a maximum of 40 points. Higher scores indicate greater perceived self-efficacy [16].

The SRQ-20 was developed by the World Health Organization to assess common mental disorders (CMDs) in low- and middle-income countries [17]. The original version contains 24 items, with the first 20 addressing nonpsychotic disorders and the last 4 addressing psychotic disorders. As the adaptation study for the Brazilian version was conducted in a PHC context, only the first 20 items are used [17]. The questionnaire includes 20 “yes” or “no” questions about emotional and physical symptoms associated with psychiatric conditions. A score of more than 8 positive responses suggests a possible mental disorder [17].

### Intervention Protocol

A nurse with experience in mental health and a PhD student in psychiatric nursing developed the intervention protocol. Two judges, including one psychologist and one occupational therapist, were chosen to review the script based on their expertise in behavioral analysis, psychological approaches, psychosocial rehabilitation, and contextual therapy training. Additionally, the program was piloted with 2 women outside the study population to test its feasibility. The researchers selected these women based on characteristics similar to the target population; however, they resided in an urban area of the city. Following the pilot tests, the content was revised to simplify the language and enhance clarity for the intended population.

The theoretical foundation of these approaches included cognitive behavioral techniques [18,19], as well as propositions and recommendations from psychiatric nursing theories [20,21].

The intervention consists of 3 modules and a workbook. The topics covered are (1) identifying emotions, (2) relationships with others, and (3) subjectivity. The topics were defined based on a situational diagnosis conducted through field visits to the rural agrarian reform community and discussions with BHU professionals responsible for the population in the area. Contextual factors such as lack of basic sanitation, financial hardship, food insecurity, unemployment, and access issues were considered, along with epidemiological and clinical factors. The program aimed to encourage reflection on the 3 previously defined topics, promoting better integration between emotional and behavioral aspects, fostering self-awareness, strengthening or enhancing internal resources for coping with daily adversities, and identifying potential to envision future possibilities.

Both the situational diagnosis and the pandemic context were considered for structure and content. Following consultations with experts (study group members with various backgrounds, including a public administrator, mental health nurses, general nurses, and psychologists), it was decided to conduct telephone-based interventions to offer guidance, active listening, structured exercises, positive reinforcement, and tailored advice.

Three 15-minute calls were made at weekly intervals. Each call included an explanation of the topic and an invitation to reflect on it using workbook activities. During the next call, participants were invited to discuss the exercise, and researchers provided at least one piece of positive feedback and one recommendation for behavioral change. Short, individualized approaches were chosen because previous studies have shown them to be factors linked to adherence [4,22].

The workbook (*Passatempo sentiMental*) contains 6 activities related to the 3 themes, combining technical and playful elements. The workbook’s development also involved collaboration with cartoonist Alexandre Beck and is currently being registered with the National Library.

### Data Analysis and Processing

The primary outcome was feasibility, and secondary outcomes included changes in psychosocial measures. We analyzed the feasibility of the program considering the 31 women who participated in at least one of the proposed approaches. The main indicator used to assess the program’s feasibility was the proportion of interested women who completed it. These aspects were analyzed using an adaptation of the procedures proposed by a group of British researchers [22], considering five aspects: (1) the number of women who accepted participation; (2) the number of participants who completed the sessions; (3) the number of participants who did not complete the sessions; (4) among those who did not complete the sessions, how many sessions they attended and their reasons for dropping out; and (5) an analysis of potential clinical and sociodemographic factors differentiating women who completed the program.

Descriptive statistics and nonparametric tests were used to analyze the overall picture of psychosocial measures (CMD symptoms, QoL domains, social support, and self-efficacy) and sociodemographic characteristics.

The Mann-Whitney *U* test was applied to compare the characteristics of women who completed the program with those who did not. Independent variables were the number of children; age (in years); and scores from QoL domains, social support, and self-efficacy. Pearson chi-square test and/or Fisher exact test were used to compare categorical variables, such as race (White, Asian, Black, or *pardo*); religion (Catholic, other, Protestant, or Evangelical); stable union (yes or no); paid work (yes or no); education level (elementary school, high school, or higher education); monthly income (less than one minimum wage, 2 to 5 minimum wages, or more than 5 minimum wages); most prevalent symptoms; and CMD indicative score (yes or no).

To assess the program’s effect, psychosocial data from the initial assessment and subsequent follow-ups were examined. The SRQ-20 questions were grouped into four dimensions, as defined by the authors based on the study’s objective: (1) somatic (questions 1, 2, 3, 7, 18, 19, and 20); (2) anxiety (questions 4, 5, and 6); (3) action and cognition (questions 8, 11, 12, 13, 14, and 16); (4) emotion and will (questions 9, 10, 15, and 17). At this stage, descriptive statistics were used to present and compare the most prevalent CMD symptoms. The Wilcoxon signed-rank test was applied to analyze differences in symptom responses between baseline and 1 month later.

The Friedman test was applied to analyze whether there were differences in psychosocial measures (CMD symptoms, QoL domains, social support, and self-efficacy) across the follow-ups (baseline, 1 week, and 1 month after the program). The tested hypothesis was that perceptions of support, QoL, and self-efficacy would increase and possible CMD symptoms would decrease after participating in the program. A significance level of 5% was adopted for all analyses. Analyses were performed using SPSS for Windows (version 25; IBM Corp).

In addition, the content of the women’s feedback on the activities was analyzed and grouped descriptively by similarity criteria.

### Ethical Considerations

All participants provided their consent by signing the informed consent formethical standards established by Resolution number 466/2012 of the Brazilian National Health Council [23]. It was approved (opinion number 67, March 10, 2021) by the research ethics committee of the School of Nursing of Ribeirão Preto, University of São Paulo. Measures were taken to ensure participants’ privacy and data confidentiality, and all data were anonymized prior to analysis. No financial compensation was provided to participants.

## Results

### Feasibility: Characteristics That Distinguished Women Who Completed All Program Activities

Participants were recruited through posters and leaflets in waiting rooms and clinics, as well as through direct approaches, with 130 women in the BHU waiting room (n=100, 76.9%) and at home (n=30, 23.1%).

**Figure 1.**
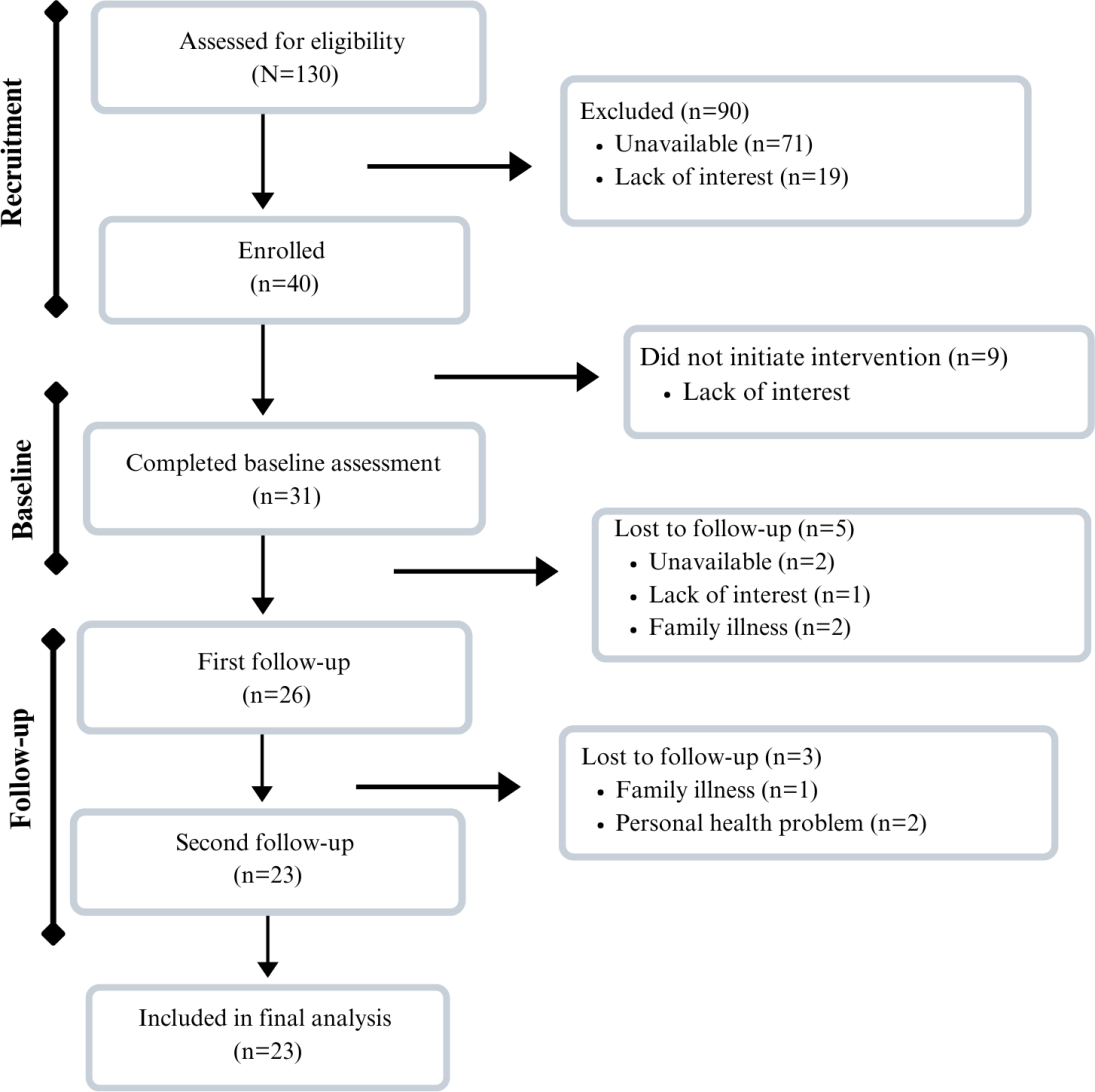
Flowchart of sample composition and reasons for dropouts after each phase of the proposed program.

Of the women who received a personal invitation to participate, 40 (30.8%) agreed to do so. They answered the pretest questionnaires and received materials to complete the activities provided in the program. However, 9 (22.5%) decided not to continue with subsequent stages of the study, resulting in a final sample size of 31 (77.5%). All participants completed the initial stage. Of the 31 women, 26 (83.9%) completed the first two stages, and 23 (74.2%) completed all three of the planned telephone-based stages (Figure 1).

Figure 1 shows that out of 31 participants, 23 (74.2%) completed all program activities. Most participants identified as Black or *pardo*, had children, had completed high school, and reported a household income of up to 2 minimum wages. There was no statistically significant difference in sociodemographic characteristics among those who completed the program and those who did not (Table 1).

**Table 1.** Sociodemographic characteristics of participants by group (completed and did not complete the program).

Variables	Completed (n=23)	Did not complete (n=8)	*P* value
Age (years), median (IQR)	42 (33-54)	40 (32-52.75)	.82
Race, n (%)			.99
Asian or White	7 (30.4)	2 (25.0)	
Black or *pardo*	16 (69.6)	6 (75.0)	
Religion, n (%)			.41
Catholic or other	7 (30.4)	4 (50.0)	
Protestant or Evangelical	16 (69.6)	4 (50.0)	
Stable union, n (%)			.58
Yes	15 (65.2)	4 (50.0)	
No	8 (34.8)	4 (50.0)	
Children, median (IQR)	2 (1-3)	2 (1.25-2.75)	.58
Yes, n (%)	20 (87.0)	8 (100.0)	
No, n (%)	3 (13.0)	0 (0.0)	
Education, n (%)			.69
Elementary school	11 (47.8)	4 (50.0)	
High school	10 (43.5)	4 (50.0)	
Higher education	2 (8.7)	0 (0.0)	
Household monthly income, n (%)			.93
Less than 1 MW^a^	9 (39.1)	3 (37.5)	
1 to 2 MWs	10 (43.5)	4 (50.0)	
2 to 5 MWs	4 (17.4)	1 (12.5)	
Paid work, n (%)			.69
Yes	9 (39.1)	4 (50.0)	
No	14 (60.9)	4 (50.0)	

a MW: minimum wage (monthly income in 2021: US $213.17).

In relation to psychosocial factors, there was a high prevalence of women with scores indicative of CMD symptoms in the entire sample (14/23, 60.9% of those who completed the program and 5/8, 62.5% of those who did not complete it). The item related to “feeling tense, nervous and worried” was the most prevalent in the pretest in both groups (Table 2 [17]). On the other hand, affirmative responses to the item “difficulty in carrying out daily life activities” were more prevalent among women who completed the program (Table 2). There was no statistically significant difference in perceived QoL, social support, and self-efficacy among the groups that completed and those that did not complete the program.

**Table 2.** Percentage of women presenting symptoms described in the Self-Reporting Questionnaire-20 (SRQ-20; adapted from the study by Mari and Williams [17]) by group (completed and did not complete the program).

SRQ-20 factors	Completed (n=23), n (%)		Did not complete (n=8), n (%)		*P* value
Somatic					
Headaches	11 (47.8)		4 (50.0)		.99
Loss of appetite	9 (39.1)		1 (12.5)		.22
Sleep difficulties	14 (60.9)		4 (50.0)		.69
Indigestion	12 (52.2)		3 (37.5)		.69
Feeling frequently tired	12 (52.2)		5 (62.5)		.70
Easily fatigued	16 (69.6)		5 (62.5)		.99
Unpleasant stomach sensations	12 (52.2)		4 (50.0)		.99
Anxiety					
Easily startled	10 (43.5)		4 (50.0)		.99
Trembling hands	7 (30.4)		5 (62.5)		.21
Feeling tense, nervous, and worried	19 (82.6)		8 (100.0)		.55
Action and cognition					
Difficulty concentrating	13 (56.5)		3 (37.5)		.43
Difficulty performing daily activities	13 (56.5)		1 (12.5)		.045^a^
Difficulty making decisions	14 (60.9)		2 (25.0)		.11
Dissatisfaction with work	6 (26.1)		2 (25.0)		.99
Feeling incapable of playing an important role in life	2 (8.7)		1 (12.5)		.99
Feelings of worthlessness	5 (21.7)		2 (25.0)		.99
Emotion and volition					
Feelings of sadness	14 (60.9)		4 (50.0)		.69
Frequent crying	7 (30.4)		2 (25.0)		.99
Loss of interest in things	12 (52.2)		5 (62.5)		.70
Suicidal thoughts	3 (13.0)		2 (25.0)		.58

a indicates statistical significance.

### Effect

Only data from the 23 women who completed all stages of the program were analyzed to determine its potential effect. A gradual increase in scores for the QoL physical, psychological, and environmental domains was observed at both follow-up assessments. However, statistically significant differences across time were found only for the physical domain, and the magnitude of change was small (Kendall *W*=0.131; *P*=.049). In contrast, CMD scores showed a gradual and statistically significant decrease over time, with a small-to-moderate effect size (Kendall *W*=0.280; *P*=.002; Table 3).

**Table 3.** Results obtained from the Friedman test at 3 assessment points (n=23).

	Baseline, median (IQR)		1 week later, median (IQR)		1 month later, median (IQR)		Chi-square (*df*)	*P* value
Quality of life domains								
Physical	57.14 (35.71-82.14)		64.29 (53.57-78.57)		71.43 (46.43-85.71)		6.0 (2)	.049^a^
Psychological	54.17 (45.83-79.17)		62.50 (50.00-79.17)		62.50 (54.17-79.17)		0.3 (2)	.85
Social relations	58.33 (41.67-75.00)		58.33 (33.33-75.00)		66.67 (41.67-83.33)		1.5 (2)	.48
Environmental	53.13 (37.50-71.88)		53.13 (40.63-71.88)		59.38 (46.88-71.88)		3.6 (2)	.16
Social support								
Satisfaction	5.33 (4.83-5.66)		5.33 (4.83-5.66)		5.17 (5.00-5.83)		0.6 (2)	.76
Number of supporters	4.00 (3.00-7.00)		4.00 (3.00-6.00)		4.00 (3.00-7.00)		5.1 (2)	.08
Self-efficacy	35.00 (27.00-37.00)		34.00 (25.00-38.00)		33.00 (24.00-38.00)		1.7 (2)	.42
Common mental disorder symptoms	9.00 (5.00-15.00)		6.00 (4.00-12.00)		5.00 (2.00-11.00)		12.9 (2)	.002^a^

a *P*<.05 indicates statistical significance.

**Figure 2.**
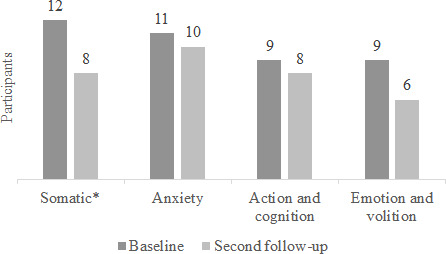
Most prevalent common mental disorder symptoms among women who completed the program at baseline and second follow-up. *P=.02.

The prevalence of CMD symptoms decreased across all dimensions, particularly in the somatic dimension, where this decrease was statistically significant (*P*=.02) (Figure 2).

### Participants’ Assessment

Regarding women’s assessment of the program’s structure, among the 23 participants, 16 (69.6%) felt the duration of the phone calls was appropriate and 13 (56.5%) said the number of calls was sufficient. All the participants rated the program positively, and 12 (52.2%) specifically highlighted the practical value of what they learned. Some expressed this utility in a more general way:

Wonderful work that helped me a lot and can help many women, mothers, wives, daughters. Thank you.

[P22]

I found the calls very interesting; I like to listen to educated people who know how to talk to us; it was very important.

[P28]

Others, on the other hand, were more specific in their comments, highlighting their favorite parts of the activities and how they applied what they learned to solve daily life issues:

It helped me a lot to understand that I am not obliged to carry the world on my shoulders and I cannot solve everything.

[P22]

I liked everything about the activity; the most interesting part was the pastime and the content; it helped me a lot because I was going through a very difficult phase and with the conversations and the notebook, I managed to organize my feelings.

[P1]

Another participant also mentioned:

It helped me see myself in a different light, to seek to love myself more, to pay more attention to myself. This pastime makes us think about what we are doing wrong with our body and mind.

[P2]

Some comments addressed the potential acceptance of the program by others:

I had never participated in an activity like this; I believe other people would enjoy these activities.

[P11]

I hadn’t participated in an activity like this before; it was the first time and, certainly, other people would like to receive this.

[P1]

In light of perceived benefits, participants recommended that the program be offered to more people:

I hope it reaches more people.

[P29]

If possible, make sure many other women know about the work*.*

[P2]

## Discussion

### Principal Findings

This study aimed to assess the feasibility and effects of a telephone intervention on the mental health of women living in a rural agrarian reform community in Brazil. High adherence to the proposed program was identified. Previous research has identified key factors that contribute to adherence, including scheduling meetings, meeting expectations regarding the study’s duration, ensuring that activities do not exceed the estimated time, and adapting session schedules to participants’ needs [4,5,22]. These factors were rigorously addressed by the program proposed in this study, and most participants positively assessed its structure and content.

Five women who did not complete the program cited illness (either their own or that of a family member for whom they were a caregiver) as the reason for their nonparticipation. This suggests that the adherence-facilitating factors noted in previous research [4,5,22], along with other strategies used in this study (eg, the possibility of rescheduling sessions and non–face-to-face modalities), were insufficient to address the specific difficulties faced by some women. This highlights the need to design interventions that address the unique challenges faced by caregivers, especially women who are culturally assigned the role of caring for their loved ones and accumulate obligations and functions [10-12], which serve as barriers to adherence to programs of this nature.

At baseline, a high prevalence of women with scores indicative of CMDs was observed, with values exceeding those reported in previous studies involving women in situations of vulnerability [24-26]. These findings suggest that women living in rural areas may face unique challenges that warrant further research to identify the actual predictors of psychological distress. This research would also help better understand the relationship between psychological distress and other well-established social determinants of health, such as socioeconomic status, race, gender, food insecurity, unemployment, agricultural labor, and limited access to health care, education, sanitation, and transportation services [10-12,27].

Additionally, prioritizing actions for women in rural communities, especially in the Brazilian context of extensive social and gender inequalities, supports the Sustainable Development Goals, which envision universal respect for human rights and human dignity, equality, and nondiscrimination based on race, ethnicity, and cultural diversity [9].

Considering PHC, nurses play a pivotal role in developing strategies to address these disparities. This is because they use essential strategies for building strong patient relationships, such as welcoming, active listening, and empathy [28], in addition to providing comprehensive care that considers the community’s biopsychosocial aspects and characteristics. In this context, nurses have significant potential to expand mental health care by creating safe spaces for emotional support and well-being for this population.

Regarding the program’s potential effects, this study observed a statistically significant reduction in CMD scores over time, with a small-to-moderate magnitude of change. Improvements were also identified in women’s perceptions of the QoL physical domain; however, the effect size was small, suggesting a modest change across assessments. These results align with propositions that emphasize the somatic dimension in manifestations of psychological distress [29,30].

Studies using interventions based on psychoeducation techniques, as well as emotional and behavioral modulation, have identified that the more patients understand their feelings and the less intrusive or aversive they consider them, the greater the reduction in somatic symptoms [31,32]. From this perspective, the program’s focus on emotional issues may have contributed to changes in symptoms related to physiological dysfunctions. Although the magnitude of change in the physical domain was small, these findings highlight the possible interplay between emotional experiences and physical manifestations of distress. These findings underscore the importance of identifying and intervening on psychosocial needs, especially in the context of PHC, where such issues are frequently overshadowed by other clinical demands.

Participants’ positive assessments regarding the activities and the emphasis placed on their importance highlight how these women need content that helps them deal with the adverse issues in their daily lives. The fact that many reported never having participated in an activity such as this raises two important issues: first, the limited access of women in general to mental health promotion, and second, the specificities related to women from agrarian reform communities.

Regarding the limited access for women in general, the findings indicated that women from the pilot test interviews (urban residents) and the study participants both reported this as their first experience with such an activity. This emphasizes the lack of mental health promotion activities for women in the general population. Concerning the specificities of women from agrarian reform communities, as they intersect various risk and vulnerability situations, it is possible that this access is even more hindered, given that local health care professionals may lack the resources or training needed to ensure access to such programs. Previous studies [10-12,33] have described issues, such as work process overload, difficulty accessing basic social rights such as health, education, and sanitation, and racial issues involving environmental, structural, and institutional racism, as significant barriers affecting the population in this study. These barriers should be further investigated to inform effective solutions.

### Limitations

This study has several limitations. First, the relatively small, context-specific sample limits the generalizability of the findings. Second, the absence of a control group precludes establishing a causal relationship between the intervention and the observed changes in mental health outcomes. Improvements identified at follow-up may have been influenced by external or contextual factors unrelated to the intervention.

Furthermore, data were predominantly self-reported and based exclusively on participants’ perceptions, which may be subject to reporting and social desirability biases. Contextual factors inherent to participants’ living conditions may also have influenced follow-up results.

As a formative study, the primary aim was to assess feasibility and preliminary effects. Further research using randomized controlled designs with larger samples is warranted to strengthen causal inferences and further assess the effectiveness of telephone-based mental health interventions for similar vulnerable populations.

Despite these limitations, the findings provide preliminary evidence supporting the feasibility of this intervention and suggest potential benefits for mental health outcomes, warranting further investigation in controlled studies.

### Conclusions

This study aimed to assess the feasibility and effects of a telephone intervention on the mental health of women from agrarian reform communities in BrazilThe study results demonstrated high adherence to the program; however, illness and caregiving responsibilities were the primary barriers to completion.

Significant improvements were identified in reducing CMD symptoms and improving physical QoL. These findings reinforce the value of integrating low-intensity psychosocial interventions into PHC for underserved populations. In this context, nurses are particularly well-positioned to lead and expand these initiatives, given their strong community engagement and essential role in health promotion, especially within the Brazilian health system.

Future research should aim to design scalable and context-sensitive approaches, such as brief structured interventions or other remote delivery strategies, that enable public policies grounded in community engagement and capable of addressing structural inequalities that limit access to mental health care for women in rural areas or other vulnerable situations.

## Abbreviations

BHU: basic health unit
CMD: common mental disorder
GSE: General Perceived Self-Efficacy Scale
PHC: primary health care
QoL: quality of life
SRQ-20: Self-Reporting Questionnaire-20
SSQ: Sarason Social Support Questionnaire
TIDIeR: Template for Intervention Description and Replication
TREND: Transparent Reporting of Evaluations with Nonrandomized Designs
WHOQOL-BREF: World Health Organization Quality of Life abbreviated version
